# Supplementary material for: Parallelized Manipulation of Adherent Living Cells by Magnetic Nanoparticles-Mediated Forces
Source: Int J Mol Sci. 2020 Sep 8;21(18):6560. doi: 10.3390/ijms21186560 (PMC7555211; doi:10.3390/ijms21186560)
Supplement: Supplementary file 1 [file ijms-21-06560-s001.pdf]

SUPLEMENTARY DATA

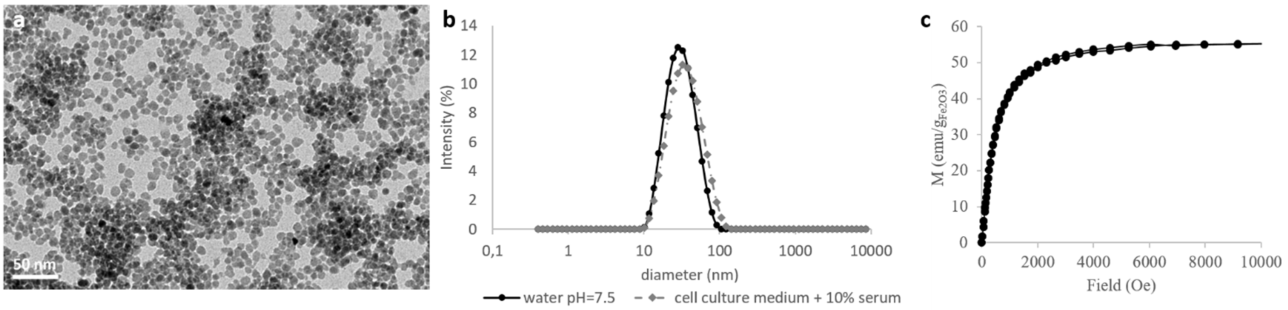

Figure S1: physical properties of the magnetic nanoparticles (MNPs) (a) Transmission electron microscopy image of the Fe_2O_3-PAA_{2k}-Rhodamine MNPs. The average physical diameter is 8.4 ± 1.9 nm. (b) Dynamic light scattering curves the Fe_2O_3-PAA_{2k}-Rhodamine MNPs in water (black circles) and in cell culture medium supplemented with 10% serum (grey diamonds). The mean hydrodynamic sizes of the particles are 27.4 nm and 30.1 in water and in cell culture medium respectively, with a polydispersity index of 0.155 and 0.223. (c) Magnetization curve of the Fe_2O_3-PAA_{2k}-Rhodamine MNPs. The MNPs are superparamagnetic with a saturation magnetization of 55.7 emu/g, and the magnetic diameter of the particles was measured with a Langevin fit to be 7.96 nm (σ=0.26).

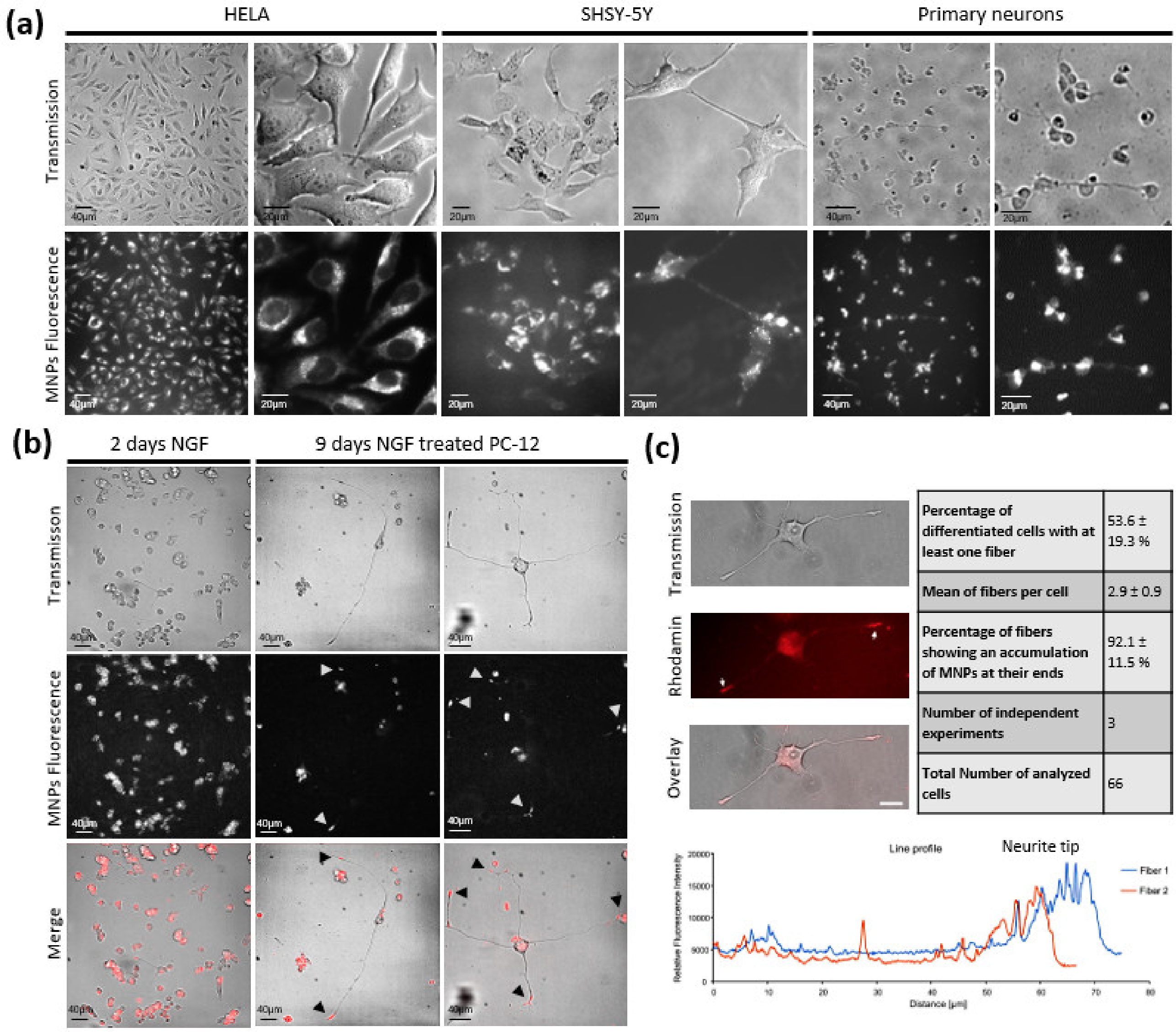

Figure S2: **uptake and distribution of MNPs inside cell models used in this study.** (a)Fluorescent and transmissionimages of Hela cells, SHSy-5Y cells and primary cortical neurons after uptake of fluorescent MNPs. The cells were incubated for18-24 hours with Fe_2O_3-PAA_{2k}-Rhodamine nanoparticles at a concentration of 10 mM or 2 mM. MNPs were successfully internalized into each cell type. (b) Fluorescent and transmission images of PC12 cells after uptake of fluorescent MNPs. The cells were incubated 24 hours with MNPs then treated for 2 or 9 days with nerve growth differentiation factor (NGF, 100 ng/ml). As for the other cell type, all PC12 cells have uptaken the MNPs. More interestingly, MNPs accumulate at the tip of the fibers in a more advanced differentiation state. (c) High magnification images of PC12 cells showing the distribution of MNPs that were taken up for 24 hours and subsequently treated with NGF (100 ng/ml) for 48 hours in differentiation medium in the absence of magnetic forces.. The table shows statistical evaluation of MNPs accumulationat the neurite tips. The graph shows the fluorescent intensity (MNPs) along the fibers of a single PC12 cell, demonstrating the accumulation at the tip.

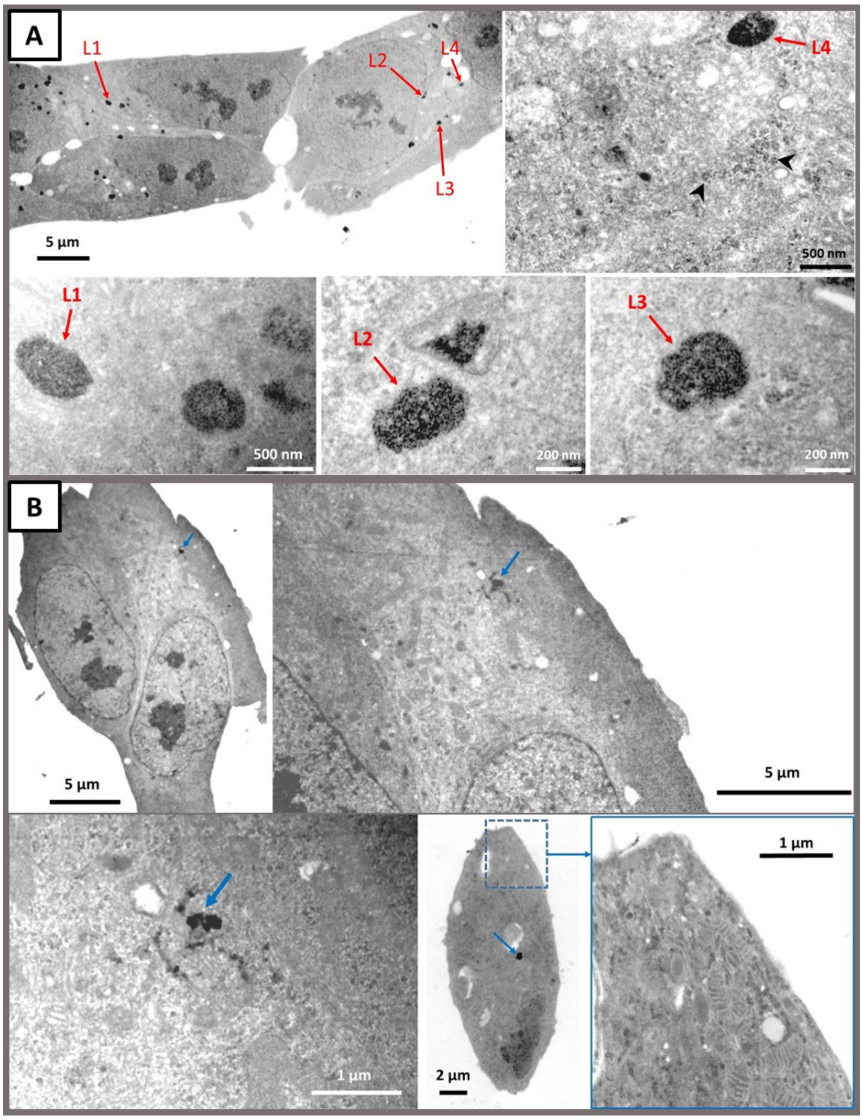

Figure S3: Bright field TEM images obtained from 100 nm thick resin embedded sections of SHSy-5Y cells, stained with 2% lead citrate, and 2% uranyl acetate. (A) Cells were incubated with Fe_2O_3-PAA_{2k}-Rhodamine MNPs for 24 hours. Red arrows indicate MNP-filled lysosomes and black arrowheads show possible MNPs in cytosol. (B) Control sample from cells incubated in the absence of MNPs. Blue arrows show lead citrate deposits.

Cell	C1	C2	C3	C4	Mean	STD
**Endosome number/section**	6	6	10	14	9	3.8
**Endosome number/cell***	48	48	80	112	**72**	**30.6**

*With section of an endosome ~500 nm and section of SHSy-5Y cell ~4 µm

Lysosome	L1	L1'	L2	L3	L4	Mean	STD
Long diameter , L (nm)	602	482	421	392	464		
Large diameter , l (nm)	553	371	220	249	272		
MNPs number/section	260	207	341	408	216		
100nm endosome volume (nm3)*	2,13E+07	1,79E+07	9,25E+06	9,75E+06	1,26E+07		
MNPs Volumetric density	1,22E-05	1,15E-05	3,68E-05	4,18E-05	1,70E-05		
Endosome volume (nm3)**	2,06E+08	1,55E+08	6,00E+07	6,33E+07	9,43E+07		
MNPs number/endosome	2515	1790	2212	2648	1611	**2155**	**448**

*Volume 100 nm endosome section=L. l. h, with h= 100 nm
**Volume endosome= 4/3. pi. L/2. l/2. ((L/2+l/2)/2). (pi/2)^3

FIGURE S4

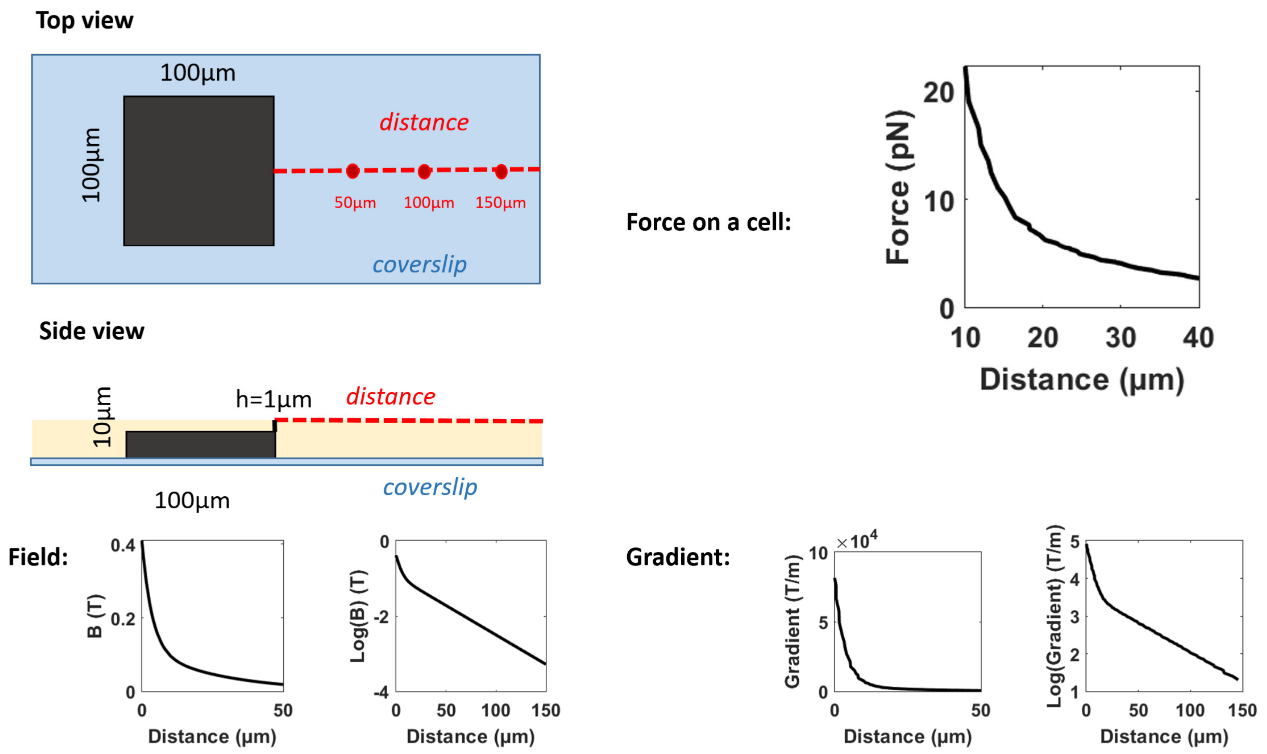

Figure S4: **Micro-pillar characterization.** The array is covered with a thin layer of biocompatible polymer (PDMS),slightly thicker than the micropillars, on top of which the cells are platted. The magnetic field (B(T)), the magnetic field gradient (T/m) and the Force per cell (pN) are plotted along a measurement region represented as a red dashed line along the surface of the PDMS layer . The plots were computed using FEM software COMSOL Multiphysics and Matlab. The magnetization curves of both the micropillar and the MNPs were taken into

account for those calculations. A typical force of 20 pN is predicted at 10 μm distance from the edge of the micropillar

FIGURE S5

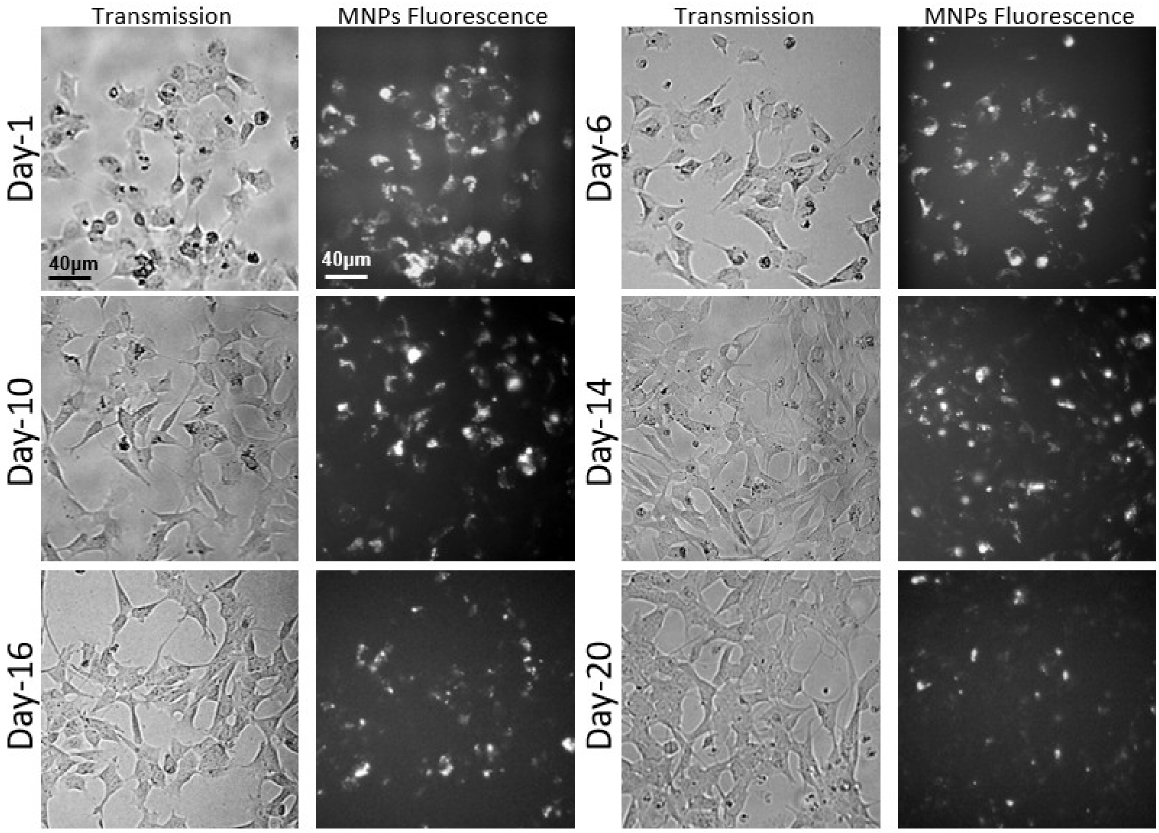

Figure S5: **MNPs Fate.** Images of SHSy-5Y cells loaded with MNPs. The MNPs were washed away using fresh medium. Cells were then cultured for 20 days under normal culture conditions.

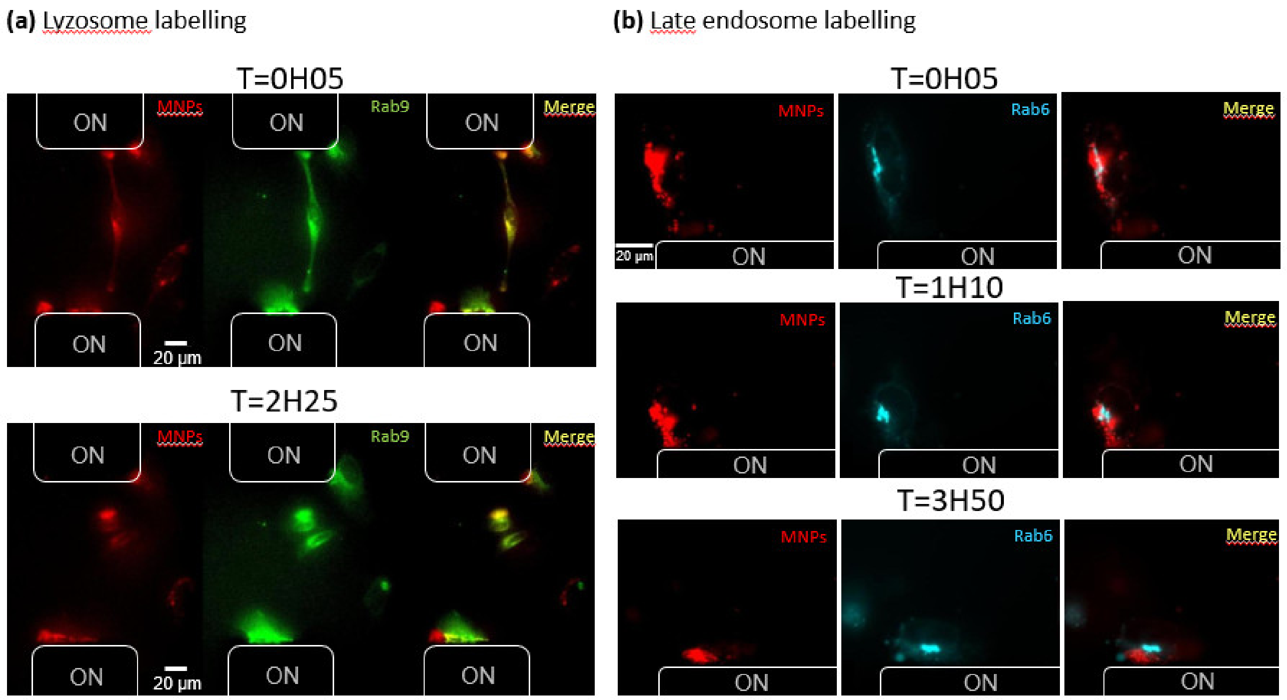

Figure S5': **a) Lysosome labeling**: Images of Hela cells previously transfected with Rab9-GFP (in green) and loaded with Fe_2O_3-PAA_{2k}-Rhodamine MNPs (in red) on the magnetic array. Pictures show different times of acquisition during 24 hours of magnetic attraction (0H05 and 2H25). Co-localization of particles and lysosomes appears in yellow**.**

b) Late endosome labeling: Images of Hela cells previously transfected with Rab6-IRFP (in blue) and loaded with $Fe_2O_3PAA_{2k}$-Rhodamine MNPs (in red) on a magnetic array at different times of magnetic attraction (0H05, 1H10 and 3H50).

VIDEOS: Voir powerpoint "Supplementary Videos"

Video S6: Parallelized magnetic control of Hela cells

Video S7: Parallelized magnetic control of SHSY-5Y cells

Video S8: Parallelized magnetic control of cortical neurons

Video S9: Parallelized magnetic control of cortical neurons

Video S10&S11: Parallelized magnetic control of PC12 cells

Video S12: Lysosome labeling of Hela cells

Video S13: Late endosome labeling of Hela cells
